# Picropodophyllotoxin Mitigates Severe Inflammation Through HMGB1 Inhibition

**DOI:** 10.3390/biom16050638

**Published:** 2026-04-24

**Authors:** Gyuri Han, Ga Eun Kim, Jong-Sup Bae

**Affiliations:** Research Institute of Pharmaceutical Sciences, Cellular & Molecular Research Institute (CMRI), College of Pharmacy, Kyungpook National University, Daegu 41566, Republic of Korea; f11074@naver.com (G.H.); rlarkdms1018@naver.com (G.E.K.)

**Keywords:** picropodophyllotoxin, barrier integrity, LPS, HMGB1, endothelium

## Abstract

Background/Objectives: Plant-derived phytochemicals are being increasingly explored for their ability to treat various illnesses, especially those affecting the vasculature. High mobility group box 1 (HMGB1) acts as a crucial mediator during the late phase of sepsis, promoting the secretion of pro-inflammatory cytokines and thereby fueling inflammation and systemic complications. Higher plasma HMGB1 levels not only hinder accurate diagnosis and prognosis but also worsen disease outcomes in inflammatory states. Picropodophyllotoxin (PPT), a key bioactive ingredient isolated from the root of Podophyllum hexandrum, has shown a range of beneficial effects, including anti-cancer and anti-proliferative actions, across several tumor types. Nevertheless, its possible involvement in HMGB1-driven severe vascular inflammation remains unexplored. The current work aimed to investigate whether PPT could influence lipopolysaccharide (LPS)-induced HMGB1 activity and its related inflammatory signaling in human umbilical vein endothelial cells (HUVECs). Methods: A combination of in vitro and in vivo approaches was used to assess the anti-inflammatory action of PPT. These included measurements of endothelial barrier function, cell survival, leukocyte attachment and migration, levels of cell adhesion molecules, and the release of pro-inflammatory factors. Both cultured human endothelial cells and mouse disease models were used to thoroughly evaluate how PPT affects HMGB1-triggered inflammatory reactions. Results: The findings showed that PPT markedly reduced HMGB1 movement from inside HUVECs to the outside, thereby limiting its release into the environment. Moreover, PPT effectively decreased neutrophil sticking and migration, lowered the appearance of HMGB1 receptors, and prevented the activation of nuclear factor-κB (NF-κB), a master switch in inflammatory signaling. At the same time, PPT treatment strongly lowered tumor necrosis factor-α (TNF-α) production, adding to its anti-inflammatory profile. Conclusions: Taken together, these results indicate that PPT potently inhibits HMGB1-driven inflammatory processes by acting at several levels of the inflammatory cascade, such as HMGB1 movement, receptor binding, NF-κB activation, and subsequent cytokine release. Therefore, PPT stands out as a hopeful therapeutic option for HMGB1-related inflammatory diseases and deserves further exploration in preclinical and clinical studies.

## 1. Introduction

In healthy conditions, high mobility group box protein 1 (HMGB1) stays inside cells; however, once it is discharged into the external environment, it becomes a strong pro-inflammatory signal [1]. Increased HMGB1 levels inside the cytoplasm signal cellular or tissue harm and serve as a warning to the innate immune system about possible infection or injury [1]. Beyond being a damage-associated molecular pattern (DAMP), HMGB1 also binds to membrane receptors such as toll-like receptors (TLR-2 and TLR-4) and the receptor for advanced glycation end-products (RAGEs) [2]. Such receptor engagement sets off downstream pathways, including nuclear factor-κB (NF-κB) and extracellular signal-regulated kinase (ERK1/2), which in turn raise the levels of cell adhesion molecules (CAMs) and encourage the creation of pro-inflammatory proteins in the surrounding matrix [2].

Because of their good safety records and wide-ranging healing promise, natural compounds from plants are being seen more and more as useful substitutes for traditional synthetic anti-inflammatory drugs [3]. Among these, picropodophyllotoxin (PPT)—a structural isomer of podophyllotoxin—is taken from the roots of Podophyllum hexandrum. PPT is a natural tyrosine kinase blocker known to suppress the insulin-like growth factor 1 receptor in several cancers, including liver cancer, non-small cell lung cancer, and muscle cancer [4,5]. Apart from its anti-cancer actions, PPT shows many biological activities, such as reducing oxidative stress and inflammation [6,7,8]. Mechanistically, PPT hinders inflammatory signaling by blocking signal transducer and activator of transcription 3 (STAT3), while at the same time triggering pro-apoptotic oxidative stress through reactive oxygen species (ROS) production and p38 mitogen-activated protein kinase (MAPK) activation. This distinct mix of anti-inflammatory and pro-oxidant effects supports its healing potential in both cancer and inflammatory diseases.

Even though anti-HMGB1 antibodies have been shown to protect against endotoxemia in mice and improve survival, their use in real-world settings is still restricted because of worries about safety and effectiveness [9]. These problems come from possibly overestimating how well they work and underestimating their toxic risks in humans, which has led to lower success in clinical settings. On the other hand, natural products from plants—which can reduce HMGB1-related inflammation without harming cells—offer a hopeful path for drug discovery. Given the safety and efficacy issues surrounding anti-HMGB1 antibodies, there is a clear need for other approaches [9]. PPT is especially attractive because of its built-in anti-inflammatory and antioxidant features, along with its good safety profile.

The importance of this work lies in showing that PPT effectively lowers HMGB1 release, keeps the endothelial barrier intact, and blocks key inflammatory pathways in both lab and animal models. By revealing how PPT reduces HMGB1-related vascular inflammation, this research sets the stage for creating new plant-based treatments for serious inflammatory diseases, meeting an urgent clinical need. With this background, we proposed that PPT could control HMGB1 production and activity under septic conditions both in vitro and in vivo. Therefore, this work aimed to see whether PPT can block endotoxin-induced HMGB1 release and its later effects on blood vessel leakage, severe inflammatory reactions, and the related molecular processes. The novelty of this study is its in-depth look at PPT as a new blocker of HMGB1-related severe inflammation in blood vessel lining cells and in live animal models.

## 2. Materials and Methods

### 2.1. Reagents

Chemicals including Picropodophyllotoxin (PPT; purity > 96%), lipopolysaccharide (LPS; serotype 0111:B4, L5293), and zingerone (ZGR; purity > 96%)—the latter utilized as a positive control per previous literature [10]—were procured from Sigma-Aldrich (St. Louis, MO, USA). This supplier also provided Evans blue dye, crystal violet, 2-mercaptoethanol, and antibiotics (penicillin G and streptomycin). Human recombinant HMGB1 was sourced from Abnova (Taipei City, Taiwan), while fetal bovine serum (FBS) and Vybrant DiD cell-staining solution were obtained from Invitrogen (Carlsbad, CA, USA). All additional reagents were of analytical grade and supplied by standard commercial vendors.

### 2.2. Cell Culture

Primary human umbilical vein endothelial cells (HUVECs) were procured from Cambrex Bio Science (Charles City, IA, USA) and maintained according to previously established protocols [11,12]. Following the procedures detailed in earlier literature [11], human neutrophils were isolated from 15 mL of whole blood donated by healthy volunteers (*n* = 5). All protocols involving human-derived materials received formal approval from the Institutional Review Board of Kyungpook National University Hospital (KNUH 2019-01-010) in Daegu, Republic of Korea.

### 2.3. Animals and Husbandry

Male C57BL/6 mice (6–7 weeks old, ~27 g) were sourced from Orient Bio Co. (Sungnam, Republic of Korea) and underwent a 12-day acclimatization period prior to the experiments. Animals were housed in groups of five per plastic cage under a regulated environment, specifically a 12 h light/dark cycle with temperatures and humidity maintained at 20–25 °C and 40–45%, respectively. Throughout the stabilization phase, mice were provided ad libitum access to standard rodent chow and water. All animal handling and experimental protocols were conducted in accordance with the ‘Guidelines for the Care and Use of Laboratory Animals’ and received approval from the Institutional Review Board of Kyungpook National University (IRB No. KNU 2024-13).

### 2.4. Cell Viability Assay

To assess cell viability, an MTT (3-(4,5-dimethylthiazol-2-yl)-2,5-diphenyltetrazolium bromide) assay was carried out as previously described [11,12]. HUVECs were placed in 96-well plates at 5 × 10^3^ cells per well and incubated for 24 h to allow attachment. After that, the cells were treated with HMGB1 (1 µg/mL) for 6 h under normal growth conditions.

### 2.5. Competitive Enzyme-Linked Immunosorbent Assay for HMGB1 and RAGE

Conditioned cells were washed 3 times with phosphate-buffered saline (PBS) and lysed with radioimmunoprecipitation assay buffer (1% protease inhibitor cocktail). Total protein was obtained from the supernatant after centrifugation (5000 rpm, 10 min), and cytosolic and nuclear fractions were extracted by using the Nuclear and Cytoplasmic Protein Extraction Kit (Thermo Fisher Scientific, Waltham, MA, USA) according to the manufacturer’s instructions. The protein concentration was determined by a BCA assay (Thermo Fisher Scientific). A competitive enzyme-linked immunosorbent assay (ELISA) was done to measure HMGB1 in the culture supernatant and to check RAGE levels, following earlier methods [13]. To measure HMGB1, HUVECs were activated with LPS (100 ng/mL) for 6 h, washed three times with PBS, and then treated with PPT for another 16 h. Conditioned medium was then collected, and HMGB1 amounts were measured using a changed competitive ELISA method as described before [13]. For RAGE measurement, HMGB1 was first mixed with RAGE with or without PPT for 12 h. Any unbound RAGE was then added to the wells coated with HMGB1. After washing three times with PBS, RAGE levels were quantified by ELISA according to earlier methods [13].

### 2.6. Permeability Assay In Vivo

Male C57BL/6 mice were anesthetized with 2% isoflurane (Forane; JW Pharmaceutical, Seoul, Republic of Korea) using an oxygen-fed inhalation system (RC2; Vetequip, Pleasanton, CA, USA). Anesthesia was induced in a breathing chamber and maintained via a face mask, ensuring stable spontaneous respiration. Following intravenous administration of HMGB1 (2 μg/mouse) for 6 h, mice were treated with PPT at doses of 0.1, 0.2, or 0.4 mg/kg. These dosages correspond to estimated peak plasma concentrations of 5, 10, and 20 μM, based on an average murine blood volume of 72 mL/kg (~2 mL for a 27 g mouse) [10]. After 16 h, Evans blue dye (1% in saline) was injected intravenously. Thirty minutes post-injection, mice were euthanized by cervical dislocation, and peritoneal fluid was harvested by lavage with 5 mL of saline. The lavage fluid was centrifuged at 200× *g* for 10 min, and the absorbance of the supernatant was measured at 650 nm. Vascular permeability was quantified as micrograms of Evans blue per peritoneal sample, utilizing a standard curve as previously described [13].

### 2.7. Permeability Assay In Vitro

The barrier function of HUVEC layers was tested with different amounts of PPT to assess its protective effect against HMGB1-induced barrier disruption in vitro. Changes in how much Evans blue-labeled albumin passed through the cell layers were measured using a modified two-chamber Transwell system, as described before [11,12]. HUVECs were added at 5 × 10^4^ cells per well into the top part of Transwell inserts with 3 μm holes (Corning, Lowell, MA, USA) and grown for 72 h to form a full layer. To induce barrier disruption, the HUVEC layers were exposed to HMGB1 (1 μg/mL) or LPS (100 ng/mL) for 6 h. After that, the cells were washed three times with PBS and then incubated with PPT at the shown amounts for another 16 h.

### 2.8. ELISA for HMGB1, Nuclear Factor-κB (NF-κB) and Tumor Necrosis Factor-α (TNF-α)

NF-κB protein levels in cell nuclei were measured with an ELISA kit (Cell Signaling Technology, Danvers, MA, USA) following the manufacturer’s instructions, while TNF-α in the conditioned medium was measured with an ELISA kit from R&D Systems (Minneapolis, MN, USA).

### 2.9. Antioxidant Effects of PPT on HUVECs

Intracellular reactive oxygen species (ROS) levels were quantified using the cell-permeable probe 2′,7′-dichlorofluorescein diacetate (DCFH-DA). Upon entering the cells, DCFH-DA is enzymatically deacetylated to a non-fluorescent intermediate, which is subsequently oxidized to the highly fluorescent 2′,7′-dichlorofluorescein (DCF) in the presence of ROS. Peroxyl radicals were generated via the thermal decomposition of 2,2′-azobis(2-amidinopropane) dihydrochloride (ABAP) at 37 °C [14,15]. For ROS detection, HUVECs (3 × 10^6^ cells/well in 24-well plates) were pretreated with PPT for 16 h, incubated with DCFH-DA for 30 min, and then challenged with 0.6 mM ABAP. Fluorescence intensity was recorded using a spectrophotometer at excitation/emission wavelengths of 485/520 nm. Additionally, oxidative stress markers—including superoxide dismutase (SOD), catalase (CAT), glutathione peroxidase (GSH-Px), and malondialdehyde (MDA)—were assessed using commercial kits (LSBio, Seattle, WA, USA) according to the manufacturer’s instructions. To induce oxidative damage, HUVECs were treated with PPT (5, 10, and 20 μM) for 16 h followed by a 24 h exposure to H_2_O_2_ (100 μM). MDA levels were measured in the culture supernatant via ELISA, while SOD, CAT, and GSH-Px activities were determined from cell lysates obtained through trypsinization and ultrasonication.

### 2.10. Cell–Cell Adhesion Assay

Human neutrophils were separated from blood and labeled with Vybrant DiD fluorescent dye as described before [11,12]. The labeled cells were resuspended at 1.5 × 10^6^ cells/mL, and 200 μL of this was added to each well of HUVEC layers. Before adding neutrophils, HUVECs were first activated with either HMGB1 (1 μg/mL) or LPS (100 ng/mL) for 6 h. After that, the HUVEC layers were washed three times with PBS and then treated with PPT for 16 h. Then, the labeled neutrophil mix was added to the HUVECs. Firmly adherent neutrophils remained on the endothelial monolayer, while non-adherent cells were removed by washing. The percentage of adherent neutrophils was calculated using:

% sticking = (signal from stuck neutrophils/signal from all neutrophils added) × 100.

### 2.11. Transendothelial Migration (TEM) Assay

Neutrophil transmigration across endothelial monolayers was evaluated using Transwell inserts (6.5 mm diameter, 8 μm pore size; Corning, Lowell, MA, USA). HUVECs were seeded at 6 × 10^4^ cells/insert and cultured for three days to achieve confluence. The endothelial layers were then stimulated with HMGB1 (1 μg/mL) or LPS (100 ng/mL) for 6 h, washed thrice with PBS, and treated with PPT for 16 h. Subsequently, freshly isolated human neutrophils were added to the upper chamber and allowed to migrate toward the HUVECs for 2 h. After incubation, non-migrated cells were removed from the upper surface of the membrane using a cotton swab. The migrated neutrophils on the underside of the filter were fixed with 8% glutaraldehyde and stained with 0.25% crystal violet in 20% methanol (*w*/*v*). Experiments were performed in duplicate. Migrated cells were quantified by capturing images and counting nine random high-power fields (HPFs) per well, with results expressed as the total number of migrated cells.

### 2.12. Expression of Cell Adhesion Molecules (CAMs) and HMGB1 Receptors

HUVEC monolayers were stimulated with HMGB1 (1 μg/mL) for 6 h, followed by three PBS washes and subsequent incubation with various concentrations of PPT (1–20 μM) for an additional 16 h. The expression levels of key adhesion molecules—specifically intercellular adhesion molecule-1 (ICAM-1), vascular cell adhesion molecule-1 (VCAM-1), and E-selectin—along with toll-like receptor 2 (TLR2), toll-like receptor 4 (TLR4), and RAGE, were quantified using commercial ELISA kits (LSBio, Seattle, WA, USA) in accordance with the manufacturer’s instructions.

### 2.13. Quantitative Real-Time PCR (qPCR)

Total RNA was taken from HUVECs using TRI Reagent (Invitrogen, Carlsbad, CA, USA) according to the manufacturer’s instructions. The RNA was then reverse-transcribed into cDNA using a PX2 Thermal Cycler (Thermo Scientific, Waltham, MA, USA). This reaction was done in 20 µL with 0.5 mg/µL oligo(dT)-adapter primers (Invitrogen) and M-MLV reverse transcriptase (Invitrogen). NF-κB and TNF-α levels were compared to β-actin as a reference. For PCR, custom primers were used: NF-κB forward: 5′-GAA TGG CTC GTC TGT AGT G-3′, reverse: 5′-TGG TAT CTG TGC TCC TCT C-3′; TNF-α forward: 5′-CCT GGT ATG AGC CCA TCT ATC-3′, reverse: 5′-AGG TTG AGG GTG TCT GAA G-3′; β-actin forward: 5′-CAA GAT CAT TGC TCC TCC TG-3′, reverse: 5′-ATC CAC ATC TGC TGG AAG G-3′. PCR steps: 94 °C for 7 min, then 40 cycles of 94 °C for 15 s and 60 °C for 45 s.

### 2.14. Statistical Analysis

All data are shown as mean ± standard deviation (SD) from at least three separate experiments. Statistics were done with SPSS software (version 16.0, SPSS Inc., Chicago, IL, USA). Differences between groups were checked by one-way analysis of variance (ANOVA) and then Tukey’s post hoc test. A *p*-value below 0.05 was seen as significant.

## 3. Results

### 3.1. PPT Suppresses LPS-Induced HMGB1 Secretion and Ameliorates HMGB1-Mediated Endothelial Barrier Disruption

**Figure 1:**
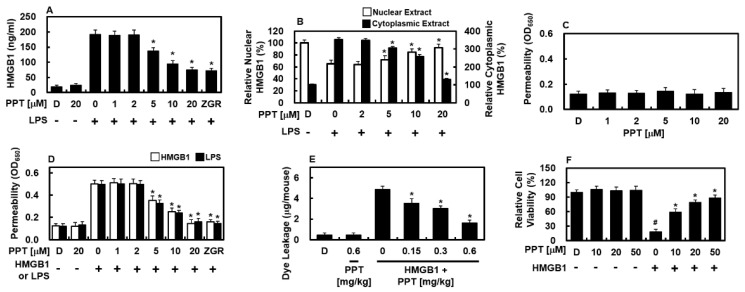
Inhibitory effects of PPT on HMGB1 release and HMGB1-caused blood vessel leakage in vitro and in vivo. (**A**) HUVECs were triggered with LPS (100 ng/mL) for 6 h before being exposed to rising levels of PPT for 16 h. HMGB1 in the conditioned medium was measured by ELISA. (**B**) HUVECs were treated with LPS (100 ng/mL) for 6 h, followed by PPT treatment (1–20 μM) for 16 h. Nuclear and cytoplasmic protein fractions were extracted, and HMGB1 levels in each fraction were measured by ELISA. (**C**) Barrier function was checked by measuring Evans blue-albumin passage through HUVEC layers after PPT treatment. (**D**) To cause leakage, HUVEC layers were first exposed to HMGB1 (1 μg/mL, white bars) or LPS (100 ng/mL, black bars) for 6 h, then treated with PPT for 16 h. (**E**) The effect of PPT on HMGB1-caused leakage in mice (*n* = 5 per group) was tested by giving HMGB1 (2 μg/mouse) through a vein. Evans blue dye in belly wash fluid was measured as micrograms per mouse. DMSO (0.5%) was used as a control. (**F**) The effect of PPT on cell viability was evaluated using the MTT assay with or without HMGB1. Data are mean ± SD from three independent experiments (*n* = 3). In panel (**A**,**B**), * *p* < 0.05 compared to LPS-treated cells (100 ng/mL) without PPT. In panel (**D**), * *p* < 0.05 compared to the respective agonist-only controls: HMGB1 alone (1 μg/mL) for white bars, and LPS alone (100 ng/mL) for black bars. In panel (**E**,**F**), * *p* < 0.05 compared to HMGB1 alone (2 μg/mouse), and # *p* < 0.05 compared to DMSO vehicle control.

Lipopolysaccharide (LPS) strongly triggers inflammation by binding to HMGB1 outside cells [16]. The HMGB1-LPS pair then moves to inside cell compartments, helping HMGB1 release and worsening body-wide inflammation, which can lead to multiple organ failure [16]. Previous study show that LPS needs HMGB1 to spread inflammatory signals, leading to treatments that target HMGB1 in sepsis models [16]. In line with earlier reports that 100 ng/mL LPS best causes HMGB1 release from endothelial cells [17], we used the same amount here (Figure 1A). Zingerone (ZGR, 20 μM) was used as a positive control to compare PPT’s anti-inflammatory strength, based on known actions [10]. To see if PPT changes HMGB1 release caused by LPS, HUVECs were treated with LPS (100 ng/mL) for 6 h and then with PPT (1–20 μM) for 16 h. As seen in Figure 1A, PPT strongly lowered HMGB1 release from HUVECs, with clear effects at levels above 5 μM. Noting that the nuclear-to-cytoplasmic translocation of HMGB1 triggers inflammatory storms via the NF-κB pathway [18,19]. Therefore, this transport mechanism represents a promising anti-inflammatory approach alongside direct expression suppression. In the nuclear protein fraction, LPS decreased the level of HMGB1, while PPT increased it (Figure 1B). The results for the cytoplasmic protein fraction were opposite of those for the nuclear protein fraction (Figure 1B). This phenomenon implies that PPT inhibits the translocation of HMGB1 from the nucleus to the cytoplasm. The ability of PPT to protect the endothelial barrier was tested in HMGB1-treated HUVECs. PPT alone did not alter barrier integrity (Figure 1C). Because HMGB1 is known to make blood vessels leaky [20,21], HUVECs were first exposed to HMGB1 (1 μg/mL) for 6 h before PPT for 16 h. As shown in Figure 1C, PPT dose-dependently reduced HMGB1-caused barrier damage. This effect was also seen when LPS was used as the trigger (Figure 1D). In live mice, PPT also lowered HMGB1-induced leakage of Evans blue dye into the belly (Figure 1E). Cell survival tests showed that PPT up to 50 μM did not harm HUVECs, and PPT also reduced HMGB1-induced cell death (Figure 1F).

### 3.2. Antioxidant Activity of PPT

**Figure 2:**
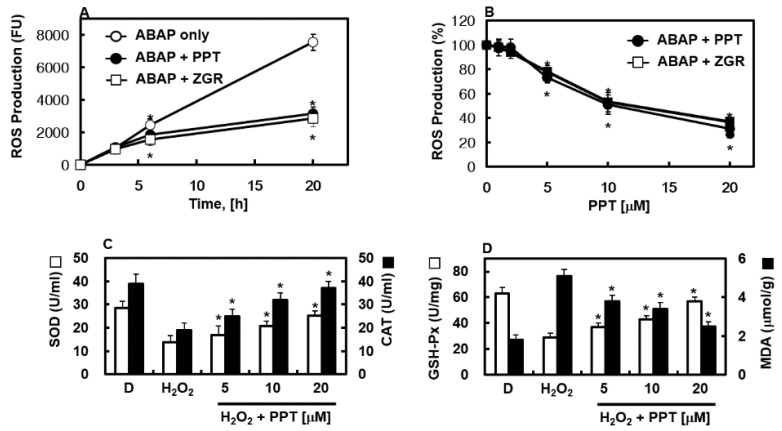
Antioxidant effects of PPT. (**A**,**B**) HUVECs were first kept with PPT (20 μM) for 16 h, then loaded with DCFH-DA for 30 min. After washing with PBS, cells were exposed to 0.6 mM ABAP to create radicals. (**A**) ROS levels were measured at different times after PPT (20 μM). (**B**) ROS production was checked 20 h after ABAP. (**C**,**D**) HUVECs were first exposed to H_2_O_2_ (100 μM) for 24 h to cause oxidative stress, then treated with rising PPT levels for another 16 h. SOD, CAT, GSH-Px, and MDA in cells were measured by ELISA. Data are mean ± SD from three experiments (*n* = 3). * *p* < 0.05 vs. ABAP alone (**A**), vs. untreated control (**B**), or vs. H_2_O_2_ alone (**C**,**D**).

We tested PPT’s ability to get into cells and fight oxidative stress caused by ABAP radicals, using a method from Wolfe et al. [22]. This approach checked PPT’s protective action through radical scavenging. Figure 2A shows that DCF oxidation in HUVECs exposed to ABAP radicals went up over time, but this was reduced by PPT. Also, Figure 2B shows that PPT lowered ROS-related fluorescence in a dose-dependent way. To learn more about PPT’s effect on oxidative harm in HUVECs, we measured SOD, CAT, GSH-Px, and MDA levels in the liquid above cells. H_2_O_2_ exposure greatly lowered SOD and GSH-Px activities compared to untreated cells, showing weaker antioxidant defenses. However, PPT at 10 and 20 μM restored SOD, CAT, and GSH-Px activities compared to H_2_O_2_-only cells. At the same time, MDA levels were much higher in H_2_O_2_-treated cells but were lowered by PPT (Figure 2C,D). All together, these results show that PPT easily enters cells and works as a strong inside-cell scavenger of hydroperoxyl radicals.

### 3.3. PPT Attenuates HMGB1-Induced Expression of Cell Adhesion Molecules and Suppresses Neutrophil Adhesion and Transmigration

**Figure 3:**
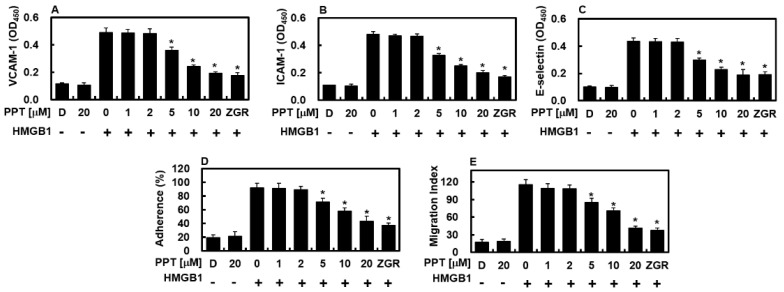
PPT blocks HMGB1-induced CAM expression, neutrophil sticking, and movement. HUVECs were treated with HMGB1 (1 μg/mL) and then with rising PPT levels for 16 h. Surface VCAM-1 (**A**), ICAM-1 (**B**), and E-selectin (**C**) were measured. (**D**) To test sticking, HUVEC layers were exposed to HMGB1 (1 μg/mL) for 6 h, then treated with PPT for 16 h, after which labeled neutrophils were added and stuck cells counted. (**E**) Neutrophil movement across HMGB1-treated HUVECs was checked after PPT for 16 h. DMSO (0.5%) was control. Data are mean ± SD from three experiments (*n* = 3). * *p* < 0.05 vs. HMGB1-only control.

HMGB1 contributes to the inflammatory response by inducing the surface expression of adhesion molecules such as ICAM-1, VCAM-1, and E-selectin on endothelial cells, thereby promoting leukocyte recruitment and extravasation into inflamed tissues [23,24]. As seen in Figure 3A–C, HMGB1 greatly raised these adhesion molecules on cell surfaces, while PPT lowered them dose-dependently. PPT’s ability to reduce CAMs suggests it interferes with HMGB1 signaling. Higher CAM levels were closely linked to more neutrophil sticking to HMGB1-treated HUVECs and later movement [23]. Of note, PPT lowered both neutrophil sticking and movement across the activated endothelium in a dose-dependent way (Figure 3D,E). In short, these results show that PPT effectively reduces HMGB1-caused neutrophil sticking and movement during inflammation.

### 3.4. PPT Suppresses HMGB1-Induced NF-κB Activation and TNF-α Production

**Figure 4:**
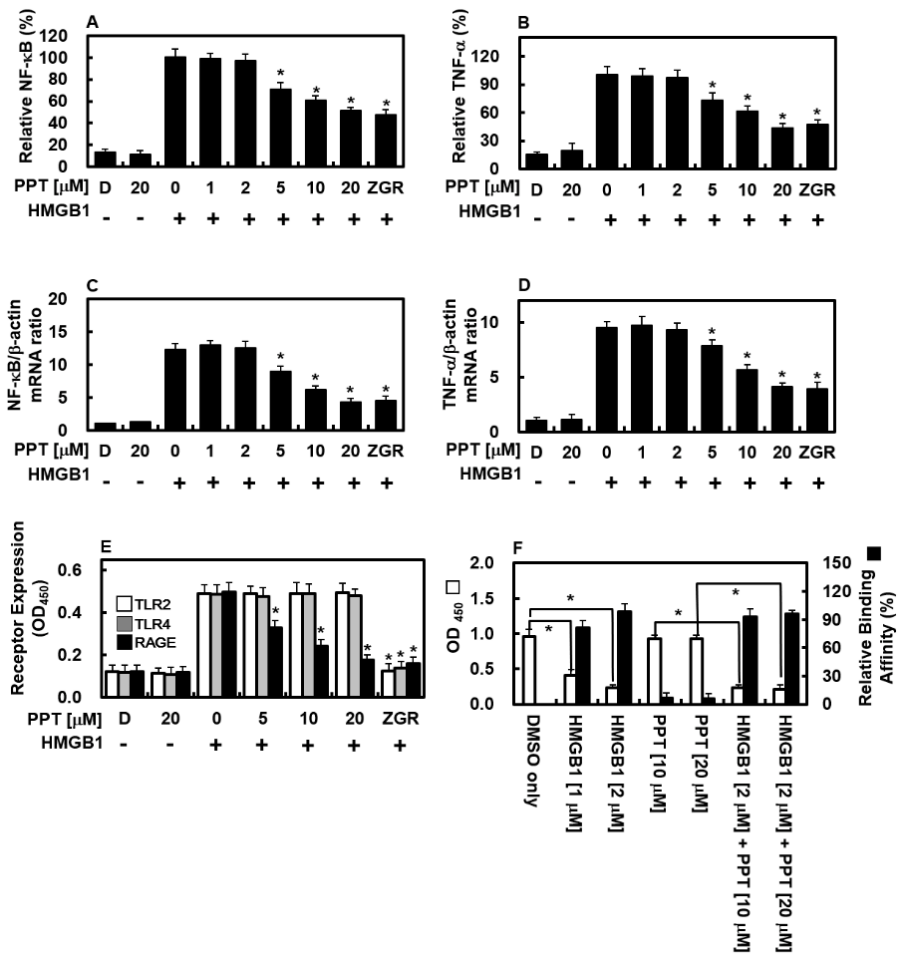
PPT blocks HMGB1-induced NF-κB, TNF-α, and pattern recognition receptors in HUVECs. (**A**–**D**) HUVECs were treated with HMGB1 (1 μg/mL) and then rising PPT for 16 h. NF-κB (**A**) and TNF-α (**B**) proteins were measured by ELISA; mRNA levels of NF-κB (**C**) and TNF-α (**D**) by qPCR. (**E**) HUVECs were exposed to HMGB1 (1 μg/mL) for 6 h, then PPT for 16 h. Surface TLR2 (white), TLR4 (gray), and RAGE (black) were measured by cell-based ELISA. (**F**) PPT’s effect on HMGB1-RAGE binding was tested by competitive ELISA. OD450 values are white bars. HMGB1 binding alone (2 μM) was set to 100%, and relative binding with PPT is black bars. DMSO (0.5%) was control. Data are mean ± SD from three experiments (*n* = 3). * *p* < 0.05 vs. HMGB1-only control (**A**–**E**).

Nuclear factor-κB (NF-κB) is a key controller of inflammation-related genes, and along with TNF-α, plays a big part in endothelial inflammatory responses [25,26]. HMGB1 is well known to activate both NF-κB and TNF-α production [18,19]. To see if PPT affects these HMGB1-driven pathways, we checked NF-κB and TNF-α in HUVECs after HMGB1. HMGB1 raised NF-κB and TNF-α levels, but PPT lowered both NF-κB (Figure 4A) and TNF-α (Figure 4B) proteins. qPCR also showed that PPT lowered NF-κB (Figure 4C) and TNF-α (Figure 4D) mRNA. Thus, PPT targets two major signaling molecules in endothelial inflammation.

### 3.5. PPT Selectively Downregulates HMGB1-Induced RAGE Expression

To see how HMGB1 affects receptor levels and how PPT changes them, we measured TLR2, TLR4, and RAGE on endothelial cells. As shown in Figure 4E, HMGB1 raised all three receptors in HUVECs. Notably, PPT selectively lowered RAGE without changing TLR2 or TLR4. To check if PPT directly blocks HMGB1 from binding to RAGE, we did a competitive ELISA. Figure 4F shows that PPT did not bind to RAGE nor stop HMGB1 from binding to RAGE. So, PPT specifically lowers HMGB1-induced RAGE levels without directly blocking the receptor binding.

## 4. Discussion

This work shows that PPT can control HMGB1 production during inflammation in both cells and animals, while also reducing HMGB1-related pro-inflammatory actions in blood vessel lining cells. This idea is backed by evidence that PPT effectively cut down HMGB1 release from HUVECs and lowered HMGB1-caused neutrophil sticking and movement toward activated endothelium. Also, PPT lowered the surface levels of HMGB1 receptors and reduced downstream signaling, as seen by less NF-κB activation and TNF-α production after HMGB1.

HMGB1 moving out of the nucleus depends on its hyperacetylation, which helps it go to the cytoplasm by blocking its return to the nucleus [27]. On the other hand, the deacetylase SIRT1 takes acetyl groups off HMGB1, making it a new target for SIRT1 [28]. Earlier studies have found that several plant compounds—such as cornuside, hederacolchiside-E, rare ginsenosides, the synthetic decursin derivative JH-4, and aloin—boost SIRT1 expression and help remove acetyls from HMGB1 [13]. Based on these, it is possible that PPT works in a similar way by raising SIRT1 and causing HMGB1 deacetylation, though more studies are needed to prove this.

Traditional Chinese herbal medicine has long been a rich source of healing substances, known for its long history, wide use, good safety, and few side effects. In this setting, natural plant compounds are being seen more as good options for treating inflammatory diseases because they work well and have known effects [29,30]. The steady chemical structure of these natural products gives a strong base for making new drugs [29]. Here, we found that PPT effectively stopped LPS-induced HMGB1 release from HUVECs and reduced the inflammatory response caused by HMGB1. PPT’s anti-inflammatory action was strongest at levels above 5 μM, which markedly reduced HMGB1 secretion, pointing to a dose-dependent effect. Because damage to the endothelial barrier is a key feature of many inflammatory diseases [31], our results highlight PPT’s ability to keep blood vessels intact by fighting HMGB1-caused leakage. This barrier-protecting role is important because keeping endothelium stable is key to limiting tissue harm and stopping inflammation from getting worse. Importantly, PPT’s ability to both lower HMGB1 release and protect the barrier suggests it acts at several points in the inflammatory network, which is better than drugs that target just one pathway. All together, these findings place PPT as a promising lead for further work on HMGB1-related blood vessel inflammation.

A key sign of a pro-inflammatory state is the high levels of adhesion molecules like ICAM-1, VCAM-1, and E-selectin on blood vessel cells [32]. The inflammatory process depends on two linked steps: white blood cells sticking firmly to damaged endothelium and then moving across it into nearby tissues [33,34]. Therefore, treatments that interfere with either sticking or movement—especially by lowering adhesion molecule levels—hold great promise for reducing inflammatory tissue damage. In our study, PPT lowered HMGB1-induced CAMs, thus blocking neutrophils from sticking to activated endothelium. This effect also led to much less neutrophil movement across the barrier. These results show that PPT fights inflammation not only by controlling HMGB1 release and receptor levels but also by directly blocking the sticking and moving steps that are central to vascular inflammation. By targeting several checkpoints in the inflammatory process—from HMGB1 release to neutrophil recruitment—PPT shows a wide-ranging action that sets it apart from standard anti-inflammatory drugs that usually hit only one target. This broad action may be helpful in treating complex inflammatory diseases where many pathways work together.

Our lab results show that PPT strongly lowers HMGB1 levels in HUVECs treated with HMGB1, which is closely linked to the NF-κB pathway and TNF-α production. Mechanistically, PPT weakened NF-κB activation, thus lowering HMGB1 levels and attenuating the downstream inflammatory response. Besides affecting HMGB1 itself, PPT also greatly lowered RAGE on the cell surface—the main receptor for HMGB1 on endothelium [34,35]—further blocking HMGB1-related inflammation. A very interesting finding is the selectivity of PPT: although it greatly lowered HMGB1-induced RAGE, it did not directly bind to RAGE or block HMGB1 from binding to it. This is important because it suggests that PPT lowers RAGE through indirect ways—possibly by changing gene activity or protein changes—rather than by blocking the receptor directly. Such a mechanism might be more specific and have fewer side effects than direct receptor blockers. All together, these results show that PPT interferes with HMGB1 signaling at several levels: by lowering HMGB1 release, blocking NF-κB, and reducing RAGE, thus creating a coordinated anti-inflammatory response without directly stopping the receptor from binding.

A particularly notable finding of this study is that PPT selectively reduced HMGB1-induced RAGE expression on endothelial cells without affecting TLR2 or TLR4 levels (Figure 4E), even though HMGB1 is known to signal through all three receptors [2]. Competitive ELISA experiments confirmed that PPT does not directly bind to RAGE nor interfere with HMGB1-RAGE binding (Figure 4F), indicating that the observed downregulation occurs through indirect mechanisms rather than direct receptor antagonism. While the precise molecular basis for this selectivity remains to be fully elucidated, several hypotheses can be proposed based on our data and the existing literature. First, differential transcriptional regulation may account for the observed selectivity. The promoter region of RAGE contains multiple functional NF-κB binding sites and is strongly dependent on NF-κB activation for both basal and inducible expression [36]. In contrast, the promoters of TLR2 and TLR4 are additionally regulated by transcription factors such as PU.1 and IRF family members, making them less exclusively reliant on NF-κB signaling [37,38]. Given that PPT significantly suppresses NF-κB activation (Figure 4A), it is plausible that RAGE expression is more vulnerable to NF-κB inhibition than TLR2 or TLR4, resulting in the selective downregulation observed. Second, distinct protein turnover rates and trafficking patterns among these receptors may contribute to the selectivity. RAGE has a relatively short half-life (approximately 4–6 h) and undergoes constitutive internalization and recycling [39]. By contrast, TLR2 and TLR4 exhibit longer surface residence times and different endosomal trafficking dynamics [40]. It is conceivable that PPT accelerates RAGE degradation or interferes with its recycling pathway without similarly affecting TLR2 or TLR4. Collectively, while the exact mechanism underlying the selective downregulation of RAGE by PPT remains to be determined, these hypotheses provide a framework for future mechanistic investigations. Studies employing promoter reporter assays, cycloheximide chase experiments, surface protein half-life measurements, and microRNA profiling will be essential to distinguish among these possibilities and to fully elucidate the molecular basis for the receptor selectivity of PPT.

High blood levels of HMGB1 have been found in patients with sudden inflammation and in animals given endotoxin [41]. This rise in HMGB1 is strongly linked to worse outcomes in serious inflammatory diseases like sepsis, where HMGB1 is a marker of how severe the disease is and risk of death [41]. As a late-stage inflammation mediator, HMGB1 is released during the later phases of endotoxemia in rodents, unlike early cytokines such as TNF-α and IL-1β [9]. Animal studies have shown that giving HMGB1 to animals causes severe gut damage and harms organ function, looking like real systemic inflammation [9,20,21]. In contrast, using anti-HMGB1 antibodies has been shown to lower sudden inflammation and improve survival in septic rodents, highlighting HMGB1’s key role in late-stage inflammatory damage [9]. All together, these findings support the idea that HMGB1 is a major driver of the late, often permanent, phase of inflammation, leading to tissue breakdown, small blood clots, and finally multiple organ failure. Importantly, our study shows that human blood vessel lining cells are a major source of HMGB1, which is released in response to harmful endotoxins and inflammatory triggers. This finding is especially important because endothelial cells sit between the blood and tissues, acting as key sensors and players in spreading body-wide inflammation. In this context, PPT’s ability to lower HMGB1 release from endothelial cells is a promising treatment strategy. By reducing this strong DAMP, PPT may break the self-feeding inflammation loop that causes tissue damage and organ failure, possibly helping in many HMGB1-related inflammatory diseases.

Beyond NF-κB activation, HMGB1 is known to engage multiple intracellular signaling cascades, including the mitogen-activated protein kinase (MAPK) family—comprising p38, c-Jun N-terminal kinase (JNK), and extracellular signal-regulated kinase (ERK)—which play critical roles in regulating pro-inflammatory cytokine production, endothelial cell activation, and leukocyte recruitment [42,43]. Upon binding to its receptors (RAGE, TLR2, and TLR4), HMGB1 triggers the sequential activation of MAPKs (MKKs), leading to phosphorylation and activation of p38, JNK, and ERK. These phosphorylated MAPKs then translocate to the nucleus and activate transcription factors such as AP-1 and NF-κB, thereby amplifying the inflammatory response [44,45]. Given our findings that PPT effectively suppresses HMGB1-induced NF-κB activation (Figure 4A) and selectively downregulates RAGE expression (Figure 4E), it is plausible that PPT may also inhibit upstream or downstream components of MAPK signaling. Several lines of evidence support this hypothesis. First, cross-talk between NF-κB and MAPK pathways is well-documented, and inhibition of one pathway often attenuates the other [46]. Second, our demonstration that PPT possesses robust antioxidant activity (Figure 2) is relevant because reactive oxygen species (ROS) serve as potent activators of p38 and JNK signaling [47]. By scavenging ROS, PPT may indirectly suppress ROS-mediated MAPK activation. Third, the selective downregulation of RAGE by PPT may reduce HMGB1-induced signaling through this receptor, which is known to couple strongly to p38 and ERK activation in endothelial cells [48]. However, we acknowledge that the current study did not directly measure the phosphorylation status of p38, JNK, or ERK following HMGB1 stimulation in the presence or absence of PPT. Such experiments—using phospho-specific antibodies in Western blot analysis or commercial ELISA kits—would be essential to definitively determine whether PPT inhibits HMGB1-induced MAPK activation. Future studies should systematically evaluate the effects of PPT on the entire MAPK signaling network, including time-course analyses of phosphorylation events and the use of specific MAPK inhibitors to identify which pathways are most critical for PPT’s anti-inflammatory actions. Given the multi-target potential of PPT suggested by our current data (NF-κB inhibition, RAGE downregulation, antioxidant activity, and HMGB1 release suppression), it is likely that PPT exerts pleiotropic effects on multiple inflammatory signaling cascades, including the MAPK family. Elucidating these pathways will be an important direction for our ongoing research.

## 5. Conclusions

The experimental data presented in this study demonstrate that PPT exerts a multifaceted protective effect against HMGB1-mediated vascular inflammation. Specifically, PPT effectively curtailed LPS-stimulated HMGB1 secretion from endothelial cells and attenuated HMGB1-induced disruption of endothelial barrier function, an effect attributed to its capacity to preserve monolayer integrity and suppress the expression of key cell adhesion molecules (CAMs). By downregulating CAMs, PPT in turn limited the adhesion and subsequent transendothelial migration of leukocytes, thereby interrupting a critical early step in the inflammatory cascade. In parallel, PPT exhibited robust antioxidant activity within HUVECs, further contributing to its anti-inflammatory effects by inhibiting HMGB1-associated activation of the NF-κB pathway and reducing the production of TNF-α—both of which serve as pivotal mediators in the propagation of vascular inflammation. Additionally, PPT selectively downregulated HMGB1-induced RAGE expression, thereby potentially diminishing the responsiveness of endothelial cells to sustained HMGB1 signaling.

Collectively, these findings position PPT as a promising candidate for the management of HMGB1-driven vascular inflammatory conditions. Its ability to simultaneously target HMGB1 release, endothelial barrier integrity, leukocyte trafficking, oxidative stress, and key inflammatory signaling pathways distinguishes it from conventional agents that typically address only one facet of the inflammatory response. Such pleiotropic activity may be particularly advantageous in complex inflammatory diseases where multiple pathways converge to sustain pathology. To advance these findings toward clinical application, future in vivo investigations employing murine models of HMGB1-induced inflammation will be essential to evaluate the therapeutic efficacy, pharmacokinetic profile, and long-term safety of PPT in the context of systemic inflammatory disorders.

## Data Availability

The original contributions presented in this study are included in the article. Further inquiries can be directed to the corresponding author [J.-S.B].

## Abbreviations

The following abbreviations are used in this manuscript:

CAM	cell adhesion molecule
CAT	catalase
DAMP	damage-associated molecular pattern
ELISA	enzyme-linked immunosorbent assay
ERK	extracellular signal-regulated kinase
GSH-Px	glutathione peroxidase
HMGB1	high mobility group box protein 1
HUVECs	human umbilical vein endothelial cells
H_2_O_2_	hydrogen peroxide
IL-1β	interleukin-1 beta
LPS	lipopolysaccharide
MTT	3-(4,5-dimethylthiazol-2-yl)-2,5-diphenyltetrazolium bromide
NF-κB	nuclear factor-kappa B
PPT	picropodophyllotoxin
RAGE	receptor for advanced glycation end-products
ROS	reactive oxygen species
SOD	superoxide dismutase
ZGR	zingerone
